# The types and effects of feedback received by emergency ambulance staff: a systematic mixed studies review with narrative synthesis

**DOI:** 10.29045/14784726.2021.3.5.4.68

**Published:** 2021-03-01

**Authors:** Caitlin Wilson

**Affiliations:** London Ambulance Service; University of Hertfordshire

## Abstract

**Aims:**

The phenomenon of feedback is well-researched within the wider healthcare context, where it is suggested that feedback can improve patient care and patient safety by enhancing clinical performance and staff mental health (Ivers et al., 2012). Within a pre-hospital context, systematic reviews have been conducted for automated feedback from defibrillators and debrief after simulation, but not on the wider concept of feedback. The aim of this systematic review was to identify, describe and synthesise the published literature on the types and effects of feedback received by emergency ambulance staff.

**Methods:**

This study is a systematic mixed studies review including empirical primary research of qualitative, quantitative and mixed-methods methodology published in peer-reviewed journals in English. Studies were included if they explored the concept of feedback as defined in this review, i.e. the systematised provision of information to emergency ambulance staff regarding their performance within pre-hospital practice and/or patient outcomes. The search strategy consisted of three facets: ambulance staff synonyms, feedback synonyms and feedback content. Databases searched on 11 June 2020 from inception were MEDLINE, EMBASE, AMED, PsycInfo, HMIC, CINAHL and Web of Science. Study quality was appraised using the Mixed Methods Appraisal Tool (Hong et al., 2018), and data were analysed using narrative synthesis guided by Popay et al. (2006) following a parallel-results convergent synthesis design.

**Results:**

The search strategy yielded 2424 articles, excluding duplicates. Seventy-eight studies met the inclusion criteria after full-text review, of which 37 only mentioned feedback as a solution to improving specific circumstances (e.g. decision-making, burnout). The remaining 41 studies consisted of: 34 interventional pre-hospital feedback studies; four non-interventional feedback studies; and three preparatory studies. The source, content and mode of pre-hospital feedback represented in the studies varied greatly and encompassed feedback on behaviour and/or feedback on outcomes of behaviour (Michie et al., 2013). The main outcome measure of included studies was quality of care (e.g. quality of CPR, adherence to guidelines) but softer measures such as staff wellbeing, professional development and clinical decision-making were also represented.

**Conclusion:**

It is anticipated that the review findings will be useful to guide the development of future pre-hospital feedback interventions, for which there is growing interest in the national and international pre-hospital setting. Further empirical research is required to explore whether the published literature reflects current pre-hospital practice.
